# Metastatic intrapulmonary hemorrhagic foci secondary to cardiac angiosarcoma: a case report

**DOI:** 10.1186/s12893-021-01132-3

**Published:** 2021-03-09

**Authors:** Yu Zheng, Guowei Che, Yun Wang

**Affiliations:** Department of Thoracic Surgery, West China Hospital, Sichuan University, West China Hospital 37 Guoxue Lane, Wuhou District, Chengdu, 610000 Sichuan China

**Keywords:** Cardiac angiosarcoma, Pulmonary metastasis, PET/CT

## Abstract

**Background:**

Cardiac angiosarcoma is a very rare malignant neoplasm, typically showing terrible prognosis. Therefore, early diagnosis is essential for efficacious intervention. Here we report a cardiac angiosarcoma with unique imaging findings because of pulmonary metastases.

**Case presentation:**

A 55-year-old man presented to our Respiratory Department because of mild morning hemoptysis for five weeks with occasional palpitations, having undergone futile antibiotic therapy for two weeks at his local hospital before admission. Symptoms of hemoptysis were alleviated with venous hemostatic drugs. ^18^F-FDG PET/CT was performed, showing a right atrial mass with multiple parenchymal nodules in lungs surrounded by ground-glass opacity, and indicated an intracardiac malignant tumor associated with pulmonary metastases, consistent with cardiothoracic CT and ultrasound. No evidence of infection or neoplasm was found using a fiberoptic bronchoscope. After multidisciplinary consultation and discussion, provisional diagnosis was established such that metastatic intrapulmonary hemorrhagic foci were secondary to intracardiac malignancy. A percutaneous biopsy from the left lung was carried out and but showed mild chronic inflammation of the lung. Therefore, urgent wedge resections for biopsy were performed from the right lung and the histopathology revealed angiosarcoma. The patient died of cardiorespiratory failure before anticancer therapy.

**Conclusions:**

Variety of clinical manifestations of cardiac angiosarcoma frequently makes its diagnosis difficult, the imaging features and epidemiology of cardiac malignancy are very significant to clinical diagnosis.

## Background

Angiosarcoma is a rare group of soft-tissue cancer with poor prognosis [1], accounting for less than 1% of all sarcomas [2]. These tumors arise from the lining of blood vessels and lymphatic vessels [3]. Usually, it can be found on the skin of head and neck [3]. Although sporadic, cardiac angiosarcoma regularly presents terrible prognosis [4, 5], particularly among those patients who cannot be subjected to treatment with radical excision [6]. Therefore, early diagnosis is very important for purposes of intervention [7].

Cardiac angiosarcoma is manifest by various non-specific clinical traits and biological behavior depending on their location and size [1, 8]. Putative diagnosis was essential for timeous intervention, and an examination of a biopsy specimen is a conclusive step in the diagnosis and treatment plan. We report an extremely rare case of right atrial angiosarcoma, and the chest CT showed multiple parenchymal nodules in lungs surrounded by ground-glass opacity because of the metastatic lesions.

## Case presentation

**Fig. 1:**
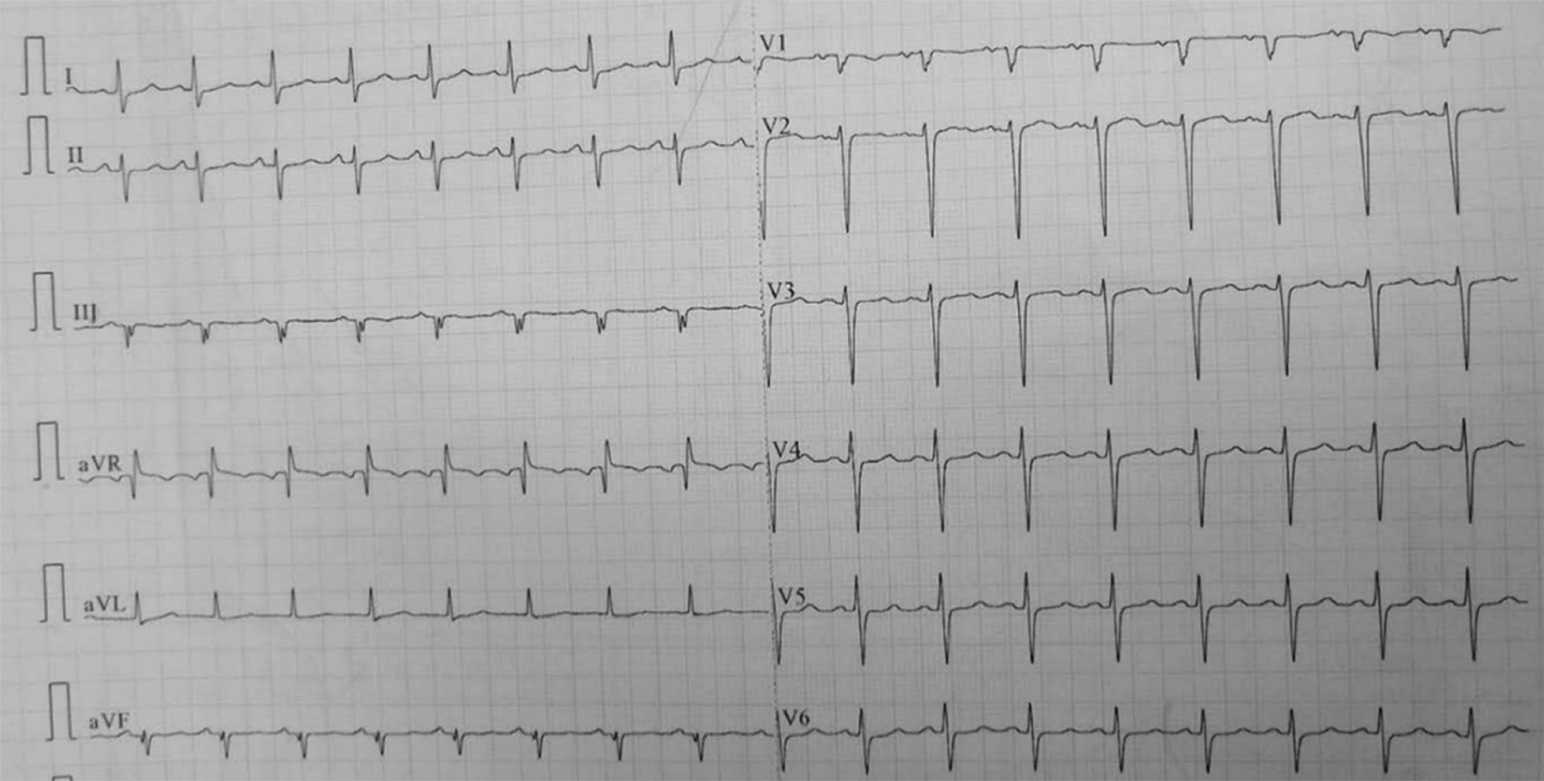
Electrocardiogram presented sinus tachycardia (103 bpm) and pseudo left-axis deviation of -82°

**Fig. 2:**
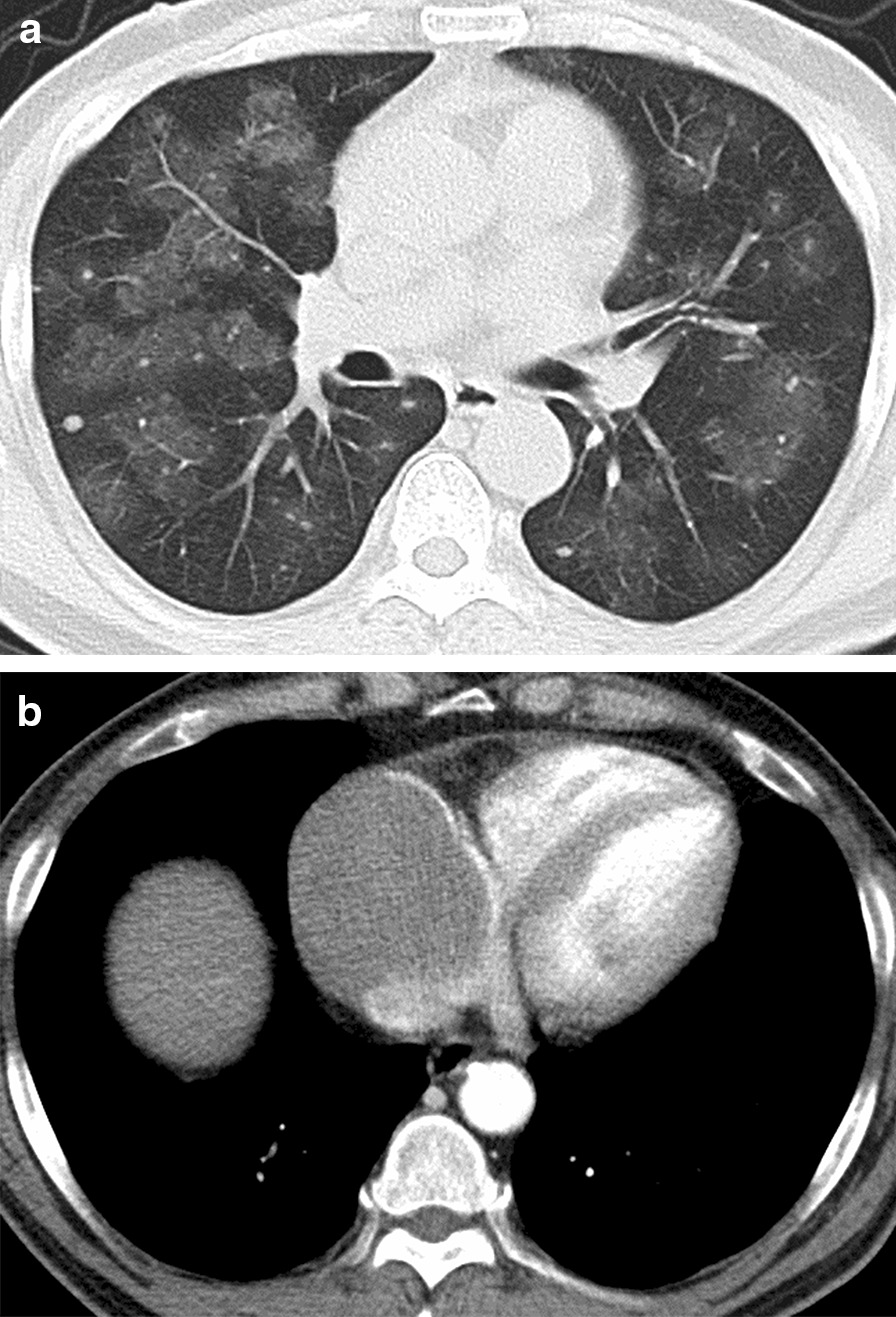
Chest CT. **a** Multiple parenchymal nodules in lungs surrounded by ground-glass opacity, **b** a mass (58 mm × 41 mm) located at the right atrium with a clear boundary

**Fig. 3:**
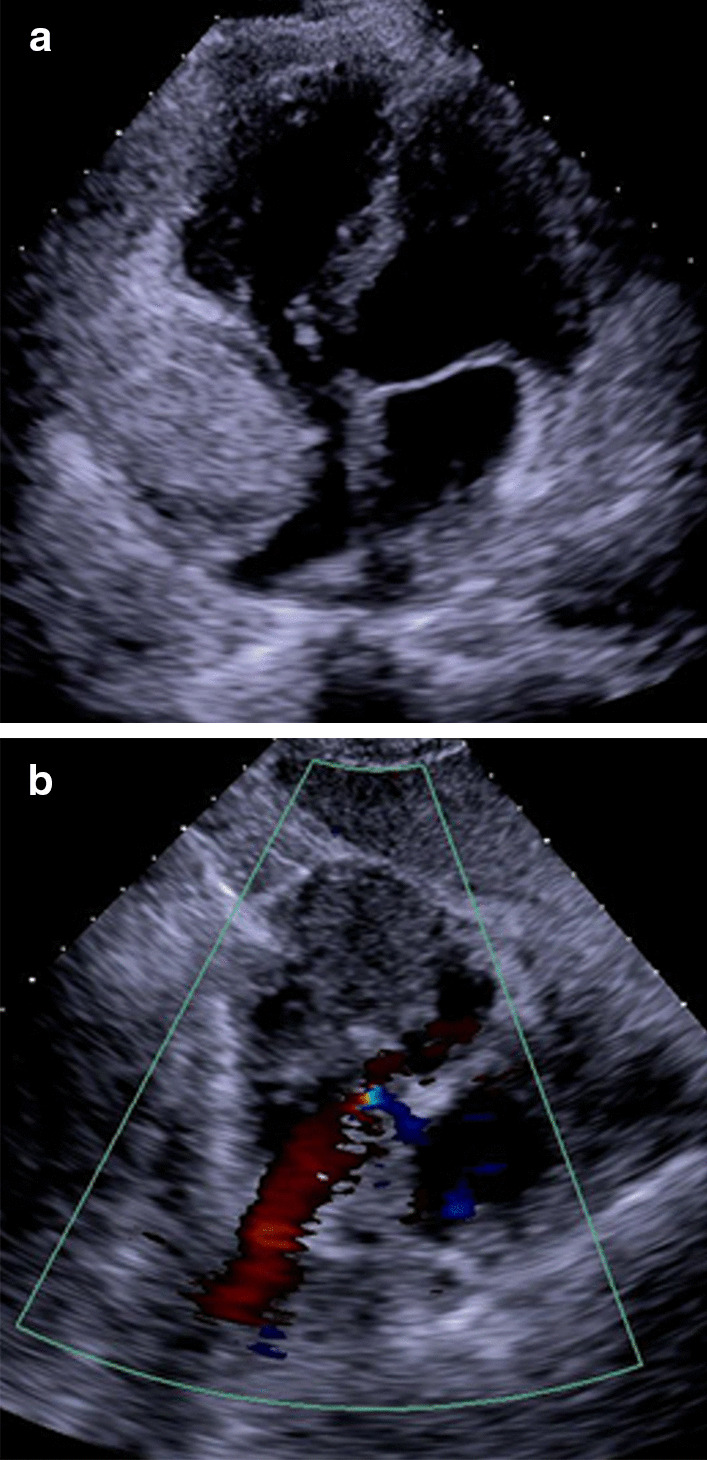
Echocardiography of heart. **a** The mass arose in the free wall of the right atrium, **b** indicating intra-neoplastic necrosis

A 55-year-old male presented to the Respiratory Department in our hospital because of mild morning hemoptysis for about five weeks, having undergone futile antibiotic therapy administered at his local hospital for two weeks before admission to our institution. Symptoms of hemoptysis were alleviated with venous hemostatic drugs in our Respiratory Department. He did not complain of fever or night sweats but presented occasional palpitations. Additionally, he has suffered from hypertension for 10 years and diabetes II for two years, respectively. A physical examination was undertaken at the time of admission, which revealed a blood pressure of 163/95 mmHg, a heart rate of 100 beats per minute, and his auscultation bilateral respiratory murmur are normal. No abnormal heart sound was detected. Laboratory investigations implied WBC, neutrophils count, and procalcitonin concentration were normal. Remarkably, D-dimer (18.46 mg/L) and fibrinogen degradation products (28.7 mg/L) were both elevated despite normal function of blood coagulation. An electrocardiogram presented sinus tachycardia (103 bpm) and pseudo left-axis deviation of -82° (Fig. 1). Chest CT exhibited multiple parenchymal nodules in lungs surrounded by ground-glass opacity (Fig. 2a), and a mass (58 mm × 41 mm) located at the right atrium with a clear boundary (Fig. 2b). Echocardiography indicated that the mass arose from the free wall of the right atrium (Fig. 3a), with uneven echoes and an anechoic area inside it (Fig. 3b), suggestive of intra-neoplastic necrosis. Left ventricle systolic function was normal (66% of ejection fraction value).

**Fig. 4:**
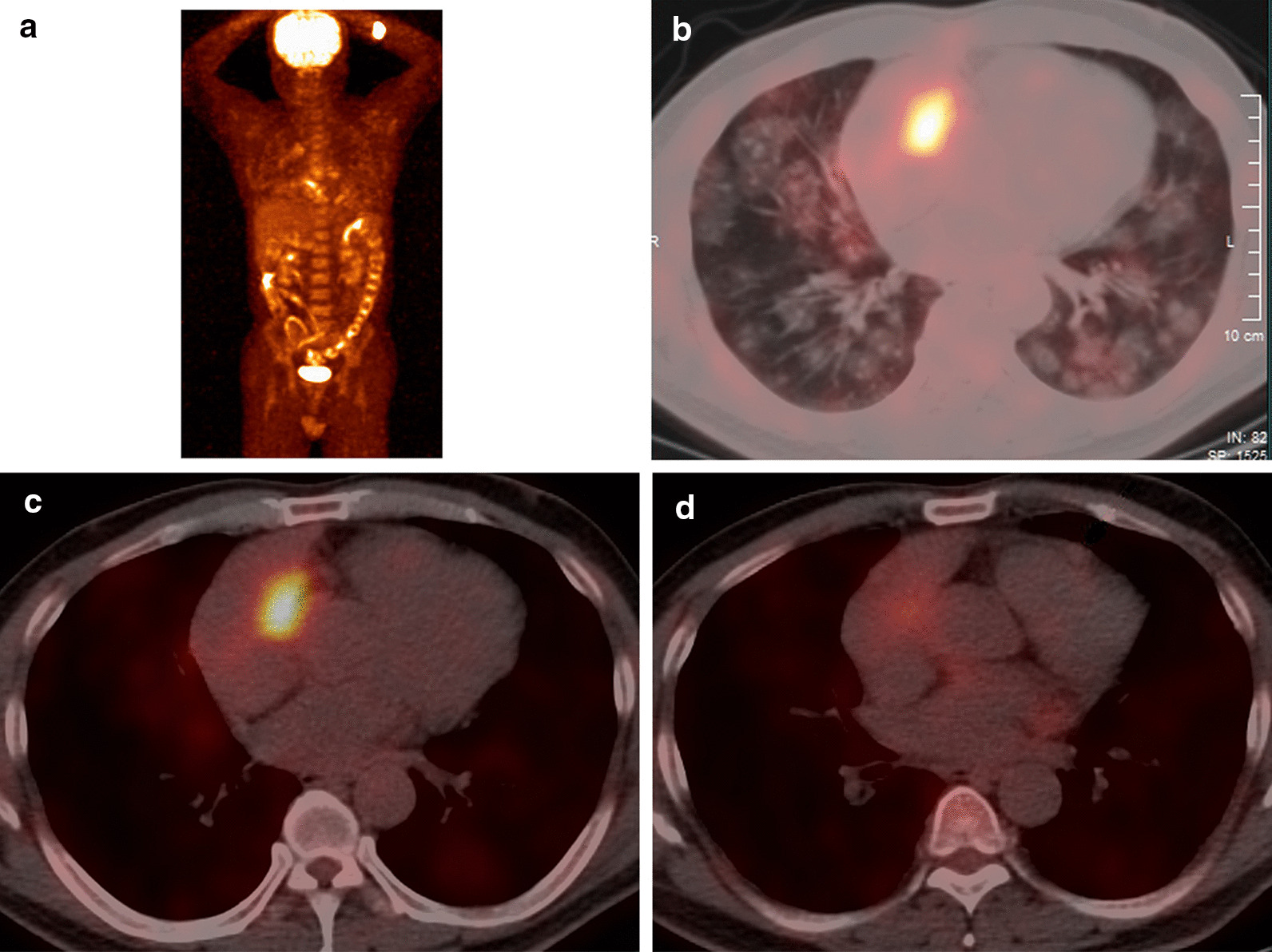
^18^F-FDG PET/CT. **a** The whole-body scan showed lesions limited to heart and lungs, **b**, **c** the chest scan showed increasing FDG uptake within the atrial lesion, lung nodules, **d** and metastatic pericardial nodules

Use of a fiberoptic bronchoscope indicated normal bronchial mucosa after removing the blood adhering to the bronchial mucosa, and the bronchoalveolar lavage fluid presented a normal cytology with no evidence of infection, consistent with laboratory tests. ^18^F-FDG PET/CT (Fig. 4) was conducted and exhibited an increase in FDG uptake within the atrial lesion, lung nodules, and pericardial nodule. Thus, a putative diagnosis was established that multiple hematogenous metastases in lung and pericardial metastases were secondary to the primary right atrial neoplasm. Thus, a pathological diagnosis is urgent.

**Fig. 5:**
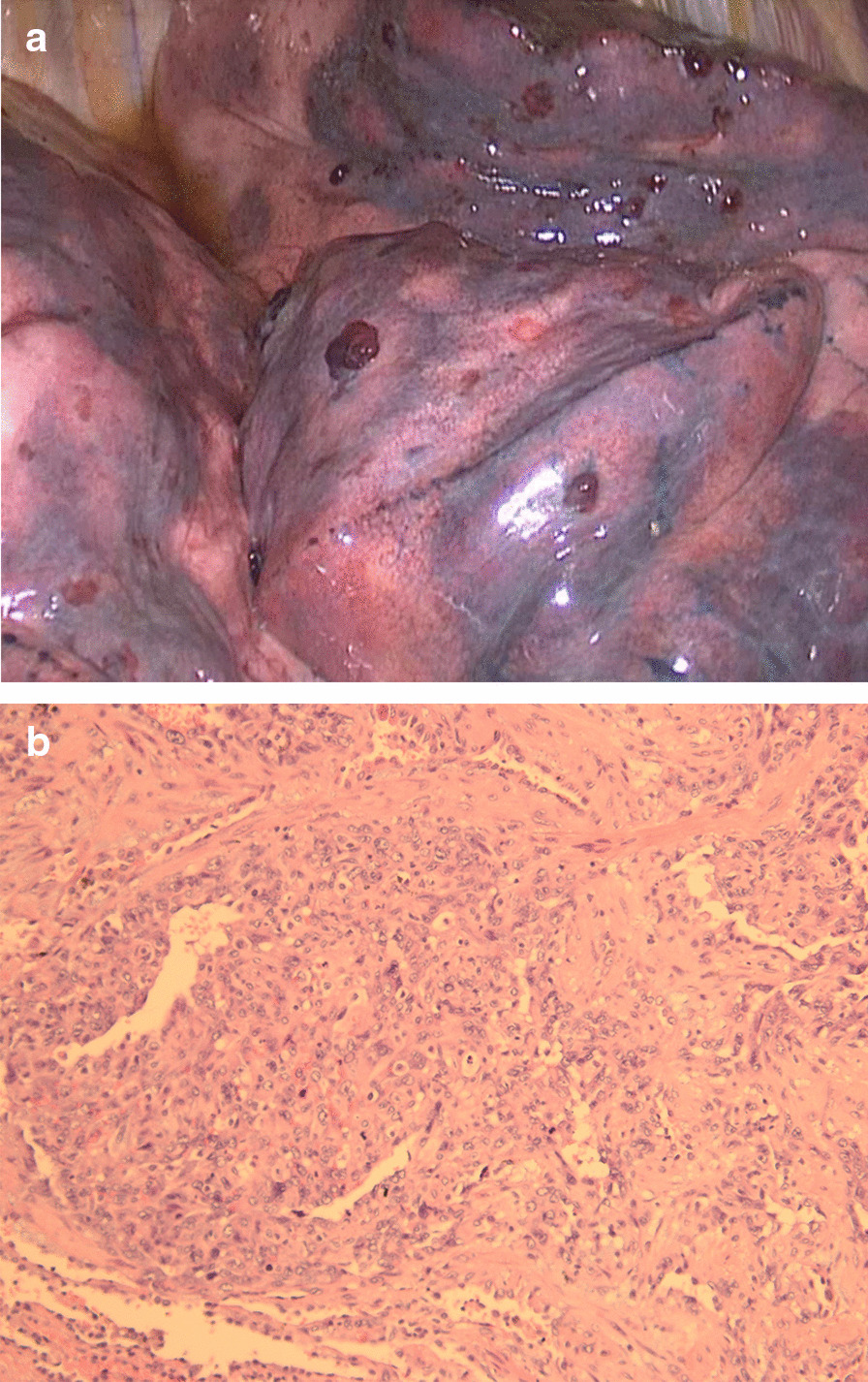
Pathology of pulmonary metastases. **a** gross examination of pulmonary metastases revealed multiple bleeding protuberances with diameters of about 5 mm to 10 mm on the lung surface, and **b** its H-E staining showed that polymorphic tumor cells with some mitoses varied in size and had vague boundary, and that irregular vascular-like fissures formed with tumor cells (×200)

A percutaneous biopsy from the left lung was performed and indicated mild chronic inflammation of the lung, accompanied by proliferation of fibroblastic tissues and alveolar epithelium cells, and by a few hemosiderin in the alveoli. Urgent wedge resections for biopsy were conducted from the right lung. The gross pathology examination showed many bleeding protuberances on the lung surface (Fig. 5a), with diameters of about 5 mm to 10 mm. The final pathologic diagnosis was angiosarcoma with H–E staining (Fig. 5b) and immunohistochemistry based on paraffin section (CD31+ , CD34+ , ERG + , CK−, EMA−, CgA−, CAMTA-1−, TFE-3−). Thus, we confirmed the diagnosis that metastatic intrapulmonary hemorrhagic foci were secondary to a right atrial angiosarcoma. The patient died of cardiorespiratory failure at one month postoperatively before chemotherapy and radiotherapy. To support an increase in the accuracy and transparency, we reported this case according to the 2013 CARE Checklist of BMC Surgery (https://www.care-statement.org/checklist).

## Discussion and conclusions

Primary cardiac malignancies are, for the most part, sarcomas [9]. Among sarcomas, angiosarcoma is most frequent (40% of sarcomas), regularly found in the right atrial chamber [10, 11]. Although angiosarcomas of the heart have been documented before [12], our case was particularly rare because it presented with a characteristic chest CT such that the parenchymal nodules were pulmonary metastases and the ground-glass opacity lesions most likely implied intrapulmonary hemorrhage.

Cardiac angiosarcomas develop various symptoms and signs depending on their location and size. Notably, the majority of patients are asymptomatic in the early stage [13]. The most common manifestation is exertional dyspnea, followed by chest pain, cough, hemoptysis, and other symptoms [14]. Our case had exclusively hemoptysis because of pulmonary metastases.

Radiological examinations are helpful for diagnosing and staging in angiosarcoma such as CT and echocardiography. As mentioned above, the chest CT findings we reported revealed a rare and interesting image characteristic because of pulmonary metastases. Moreover, laboratory tests also contribute to the identification of this disease, especially when hemoptysis occurs. In the present patient, increasing level of D-dimer and fibrinogen degradation product implied the possibility of hemorrhage in lungs. Sputum examination and fiberoptic bronchoscopy should be performed for differential diagnosis from infection. Notably, malignancy is possibly overlooked when patients develop neoplasm associated infection of heart and lungs. ^18^F-FDG PET/CT is useful for differentiation before biopsy [12]. Given that his malignant lesions were limited to the right heart, pericardium, and the lungs according to the PET/CT, combined with the features of pulmonary circulation, we confirmed the primary right atrial angiosarcoma developed hematogenous metastasis only in the lungs owing to the barrier of pulmonary capillaries. Blood supply for pericardium is from systemic circulation, so pericardial nodules arose due to invasion and dissemination.

For cardiac malignancy, pathological diagnosis is the gold standard based on immunohistochemistry as evidence for chemotherapy and radiotherapy. Urgent biopsy must be performed from primary tumors or from metastatic lesions when curative operation is not considered. Above all, clinicians should make as early a diagnosis as possible based on the clinical manifestations and imaging examination and epidemiology in these cardiac malignancies.

## Data Availability

The datasets used and/or analyzed during the current study are available from the corresponding author on reasonable request.

## Abbreviations

^18^F-FDG: ^18^F-flurodeoxyglucose
PET: Positron emission tomography
CT: Computed tomography
WBC: White blood cell count
